# Diagnostic performance of routine platelet indices and novel hemogram-derived composite biomarkers in adult sepsis

**DOI:** 10.3389/fmed.2026.1953501

**Published:** 2026-10-29

**Authors:** Cemile Uyar, Emel Koçak, Öznur Ak, Sevil Alkan, Fatmagül Can, Pınar Korkmaz, Serpil Genç

**Affiliations:** 1 Department of Infectious Diseases and Clinical Microbiology, Faculty of Medicine, Kütahya University of Health Sciences, Kütahya, Turkey; 2 Department of Medical Biochemistry, Faculty of Medicine, Kütahya University of Health Sciences, Kütahya, Turkey; 3 Department of Infectious Diseases and Clinical Microbiology, Faculty of Medicine, Çanakkale Onsekiz Mart University, Çanakkale, Turkey; 4 Department of Medical Microbiology, Faculty of Medicine, Kütahya University of Health Sciences, Kütahya, Turkey

**Keywords:** sepsis, diagnosis, biomarkers, platelet indices, plateletcrit, composite biomarkers

## Abstract

**Aim:**

Sepsis is a serious infectious condition that can lead to multiple organ failure and can be fatal if not diagnosed and treated early. Although many parameters have been evaluated in the early diagnosis of sepsis, platelet-related factors have been studied to a limited extent. The aim of this study was to evaluate conventional platelet indices and explore the diagnostic performance of hemogram-derived composite indices, including WBC × PDW, in adult sepsis.

**Methods:**

This retrospective study included 101 patients diagnosed with sepsis according to the Sepsis-3 criteria and 100 healthy control patients between 1 July 2023 and 31 December 2023. Laboratory parameters thought to be associated with inflammation were compared between the groups.

**Results:**

In the sepsis group, a significant increase in white blood cell (WBC) and neutrophil counts was observed compared to the control group, while hemoglobin, lymphocyte count, platelet count (PLT), and plateletcrit (PCT) values decreased significantly. Mean platelet volume (MPV) and platelet distribution width (PDW) values were found to be significantly higher in sepsis group. Furthermore, PDW showed the strongest correlation with procalcitonin and inflammatory parameters. According to the ROC analysis, in the evaluation of the diagnostic performance of conventional platelet indices (such as MPV and PDW) and combined ratios (such as MPV/PLT and PDW/PLT), the WBC × PDW index demonstrated the strongest discriminatory value (AUC = 0.815).

**Conclusion:**

While platelet counts decreased in patients with sepsis, MPV and PDW values increased significantly. New composite and ratio indices derived from the complete blood count [MPV/PLT [AUC = 0.712], PDW/PLT [AUC = 0.748], and the WBC × PDW index [AUC = 0.815]] demonstrated superior diagnostic performance in distinguishing sepsis from healthy controls.

## Introduction

Sepsis, a systemic inflammatory response triggered by infection, arises from the individual's response to the infection. Despite advances in antimicrobial treatments and diagnostic methods, sepsis leads to high mortality (1). The clinical manifestations of sepsis vary considerably, ranging from mild reactions to life-threatening severity. Sepsis causes a complex interaction between the host and infectious agents, leading to the activation of numerous inflammatory pathways, including the coagulation system, complement system, and cytokine networks (2). Whilst the process is initiated by microorganisms or their toxins, the body's excessive response leads to widespread inflammation, multiple organ dysfunction and organ failure (3, 4).

Cost-effective laboratory tests used in the rapid assessment of sepsis are very important in clinical practice; white blood cell count, C-reactive protein (CRP), erythrocyte sedimentation rate (ESR) and procalcitonin are the most common acute phase reactants and are key biomarkers for sepsis diagnosis and monitoring (5). In addition, platelet count, mean platelet volume (MPV) and platelet distribution width (PDW) have been found to be effective in this regard (5). In these patients, an increase in MPV and PDW levels has been observed alongside a decrease in platelet count. It is thought that these findings stem from endothelial damage, cytokine production and bone marrow suppression in patients diagnosed with sepsis (6, 7).

Platelets regulate systemic inflammation, immune responses, angiogenesis and wound healing through their interactions with leukocytes and the substances they release (8). Automated blood counts derive essential platelet indices (PIs) reflecting platelet morphology and activation: platelet count (PLT), MPV, PDW, plateletcrit (PCT), platelet large cell ratio (P-LCR), and platelet large cell count (P-LCC) (9–12). Physiologically, there is a direct proportional relationship between MPV and PDW (10, 11). MPV reflects the rate of platelet production and activation, whereas PDW measures size variability and reflects morphological heterogeneity (11). PCT represents platelet volume relative to total blood volume (normal range: 0.22%–0.24%) and is derived from PLT and MPV (11, 12). P-LCR and P-LCC represent, respectively, the percentage and count of platelets larger than 12 fL; P-LCR (normal range: 15%–35%) is also used to monitor platelet activity (13, 14). Elevated MPV typically indicates regenerative thrombocytopenia associated with peripheral destruction or consumption (15). However, MPV acts as both a positive and negative acute-phase reactant; whilst high MPV is associated with active inflammation, severe and high-grade inflammation can lower MPV due to the intensive consumption of large platelets in inflammatory sites (16). Critical conditions such as sepsis, ARDS, MI, and pulmonary embolism are associated with poor prognosis and high mortality (17). In severe sepsis, the MPV/PLT ratio serves as a promising prognostic indicator (18, 19). Particularly in septic shock, elevated MPV, PDW and P-LCR levels, alongside reduced PLT and PCT, indicate increased mortality and a poor prognosis (15).

Traditional markers such as CRP and procalcitonin, which are used for early diagnosis and prognosis in sepsis, are sometimes inadequate. Given the limited number of studies on this topic in the literature, this study was designed to evaluate the role of PIs—which reflect inflammatory and thrombotic processes in sepsis and are rapid, inexpensive and readily available—as a diagnostic marker in sepsis.

## Methodology

This study was designed as a single-center retrospective case-control study and it was conducted at Kütahya Sağlık Bilimleri Üniversitesi Evliya Çelebi Training and Research Hospital. The study included 101 adult patients (sepsis group) who were hospitalised between July 1 and December 31, 2023, and diagnosed with sepsis, as well as 100 healthy individuals (control group) with negative infection markers. Data on the included patients were retrospectively reviewed by the principal investigator through the hospital's automation system records.

### Inclusion criteria

Patients meeting the inclusion criteria were individuals over the age of 18 who were diagnosed with sepsis by infectious disease specialists based on Sepsis-3 criteria (20) (defined as an increase of ≥2 points in the acute SOFA score—assuming a baseline of zero in patients without pre-existing organ dysfunction—in the presence of documented or suspected infection). The patients' diagnoses were confirmed by growth in microbiological cultures (blood, urine, sputum, etc.) or by radiological imaging findings consistent with an infectious focus. Patient data include complete blood count (CBC) and inflammatory markers (CRP and procalcitonin).

No distinction was made between community-acquired sepsis cases and those that developed sepsis during hospitalization. All blood samples were selected based on values recorded immediately prior to the initiation of antibiotic therapy and at the time the sepsis diagnosis was established.

### Exclusion criteria

Pregnant women, patients < 18 age, patients with autoimmune diseases, patients with platelet or coagulation disorders, patients undergoing chemotherapy or radiotherapy for malignancies affecting the coagulation system or platelets, patients with known haematological disorders, medications that alter clotting or platelet function (warfarin, heparin, etc.), patients who have received a platelet transfusion or a transfusion of fresh frozen plasma or concentrated blood clotting products within the previous 10 days, and patients with liver failure were excluded.

### Control group

The control group was selected from healthy individuals aged ≥18, with similar gender to the patient group, and negative infection parameters.

### Data collection tools and characteristics

Demographic data were collected by the responsible researcher. In patients diagnosed with sepsis, necessary tests such as CRP, procalcitonin, complete blood count (CBC), platelet parameters, and blood cultures were performed in the medical biochemistry and medical microbiology laboratories to establish the diagnosis of infection and sepsis and to exclude infection status. Platelet parameters were statistically evaluated as a subset during the CBC analysis. All data from CRP, procalcitonin, CBC, platelet parameters, and blood cultures for patients diagnosed with sepsis were statistically analyzed based on the data obtained from the medical biochemistry and medical microbiology laboratories.

### Ethical approval

Ethics approval has been obtained from the our Health Sciences Rectorship Non-Invasive Clinical Research Ethics Committee with decision number 2024/08-04, dated 25.06.2024.

### Laboratory tests

The complete blood count was analysed using the Mindray BC-6800 automated haematology analyser (Mindray Bio-Medical Electronics Co., Ltd, Shenzhen, China) with original reagents. Serum CRP levels were measured using the Beckman Coulter AU5800 analyser (Beckman Coulter, Miami, FL, USA) with original reagents. Serum procalcitonin levels were measured using the Maglumi 2000 analyser (SNIBE, Shenzhen, China) with original reagents.

### Calculation of composite indices

The WBC × PDW index was calculated as the product of the total leukocyte count and the platelet distribution width to evaluate combined inflammatory and platelet-related diagnostic performance.

To optimize the numerical range for clinical interpretation and ROC curve modeling, the product of the total leukocyte count 10^3^/μl and the platelet distribution width (PDW, fL) was divided by 6.

$$\mathrm{WBC}\times \mathrm{PDW}\;\mathrm{Index}=\frac{\mathrm{WBC}\;(10^{3}/\mu l)\;X\;\mathrm{PDW}\;(\mathrm{fL})}{6}$$

### Statistical methods

The Kolmogorov–Smirnov test was used to assess the normality of variable distributions. The two-sample t-test was used to compare continuous variables showing a normal distribution between groups. Continuous variables showing a normal distribution are presented as mean ± standard deviation. The Mann–Whitney U test was used to compare continuous variables not showing a normal distribution between groups. The median and interquartile range (IQR) were used to present continuous data that did not follow a normal distribution. Categorical variables were compared using Fisher's exact test. Categorical variables were presented as counts. As the data did not follow a normal distribution, correlation analyses were performed using Spearman's correlation analysis. Receiver operating characteristic (ROC) curve analysis was used to identify the most accurate diagnostic biomarker for distinguishing between sepsis and normal conditions. A *p*-value <0.05 was considered statistically significant. Statistical analyses were performed using GraphPad Prism version 6.05 (GraphPad Software, Inc., CA, USA).

In order to minimise the confounding effect of the significant difference in chronological age between the sepsis patient group and the healthy control cohort, an age-adjusted multivariate logistic regression model was applied. This statistical adjustment ensured that the significant differences observed in platelet parameters (MPV, PDW and PCT) were associated with the clinical status of sepsis independently, rather than being a result of the age difference.

## Results

The study included 100 healthy controls and 101 patients diagnosed with sepsis. Of the 101 sepsis patients included in the study, 90 (89.1%) were admitted to the intensive care unit (ICU), and 54 (53.5%) developed septic shock. Septic shock was defined as the need for vasopressor agents to maintain a mean arterial pressure (MAP) of 65 mmHg despite adequate fluid resuscitation, accompanied by a serum lactate level >2 mmol/L. When evaluated according to the source of infection, the most common causes were found to be catheter-related infections (*n* = 36; 35.6%), pneumonia (*n* = 34; 33.7%), urinary tract infections (*n* = 25; 24.8%), intra-abdominal infections (*n* = 4; 4.0%), and soft tissue infections (*n* = 2; 2.0%), respectively.

The mean age of the patient group (66 ± 14 years) was significantly higher than the mean age of the control group (49 ± 21 years) (*p* < 0.0001). Gender distribution was similar between the two groups (*p* = 0.162). Table 1 summarises the demographic characteristics and laboratory findings of patients with sepsis compared to the control group.

**Table 1:** Demographic characteristics and laboratory findings of patients with sepsis and control groups.

Variables	Control (*n* = 100)	Patient (*n* = 101)	*P*-value
Age (year)	49 ± 21	66 ± 14	<0.0001
Gender			
Female	58	45	0.162
Male	42	56	
WBC (10^3^/uL)	6.92 (6.03–8.28)	12.43 (9.58–16.80)	<0.0001
NEU (10^3^/uL)	4.43 (3.39–5.18)	9.94 (7.19–13.42)	<0.0001
LYMPH (10^3^/uL)	2.02 (1.50–2.40)	1.04 (0.70–1.92)	<0.0001
HGB (g/dL)	13.2 (12.3–15.0)	10.9 (9.4–13.0)	<0.0001
RDW-CV	13.0 (13.0–14.0)	14.3 (13.0–16.4)	0.0001
RDW-SD	42.0 (40.0–44.0)	45.0 (42.0–50.5)	<0.0001
PLT (10^3^/uL)	262.5 (228.0–315.5)	229.0 (176.5–312.0)	0.03
MPV (fL)	9.0 (8.2–10.0)	9.4 (9.0–10.0)	0.04
PDW	16.0 (15.0–16.0)	16.0 (15.8–16.1)	<0.0001
PCT (%)	0.26 (0.22–0.29)	0.23 (0.18–0.30)	0.03

*WBC, White blood cell; NEU, Neutrophil; LYMPH, Lymphocyte; HGB, Hemoglobin; RDW-CV, Red cell distrubition width - coefficient variation; RDW-SD, Red cell distrubition width - standart deviation; PLT, Platelet; MPV, Mean platelet volume; PDW, Platelet distrubition width; PCT, Plateletcrit. Normally distributed data were expressed as mean ± standard deviation (SD) and not normally distributed data were expressed as median and interquartile range (IQRs). The comparisons of data between groups were analyzed with the *two independent-sample t-test* and the *Mann–Whitney U test* according to the distribution of data. Categorical variables were compared by *Fisher's exact test*. Categorical variables were presented as a count. A *P*-value <0.05 was considered statistically significant.

Table 1 summarises the comparison of demographic and laboratory data of patients diagnosed with sepsis and a control group. In the sepsis group, white blood cell count (WBC) (*p* < 0.0001), neutrophil count (NEU) (*p* < 0.0001), RDW-CV (*p* = 0.000), RDW-SD (*p* < 0.0001), MPV (*p* = 0.04), and PDW (*p* < 0.0001) values were statistically significantly higher; and lymphocyte (LYMPH) (*p* < 0.0001), HGB (*p* < 0.0001), PLT (*p* = 0.03), and PCT (*p* = 0.03) levels were significantly lower compared to the control group (Table 1). Although the median PDW values for the sepsis and control groups exhibited a narrow numerical range (16.0 vs. 16.0) with overlapping interquartile ranges, the highly significant *p*-value obtained (*p* < 0.0001) reflects a distinct shift in cumulative distribution as assessed by non-parametric tests. This indicates a significant difference in terms of rank order rather than a large difference in medians.

Table 2 summarizes the relationships between CRP, procalcitonin, WBC, NEU, LYMPH, and various PIs in patients with sepsis. According to this table, procalcitonin, WBC, and NEU values show a negative correlation with PLT, while they show a positive correlation with MPV and PDW. Lymphocyte counts show a positive correlation with PLT and a negative correlation with PDW; lymphocytes have no statistically significant effect on MPV. PCT did not show significant correlations with the other parameters, except for a significant positive correlation with lymphocytes (*p* = 0.0003).

**Table 2:** The relationship between CRP, procalcitonin, WBC, NEU, LYMPH and platelet indices of the patients with sepsis.

Platelet Parameters	CRP (mg/L)		Procalcitonin (ng/mL)		WBC (10^3^/uL)		NEU		LYMPH	
	r	P	r	P	r	P	r	P	r	P
PLT (10^3^/uL)	−0.047	0.643	−0.224	0.02*	−0.226	0.02*	−0.204	0.04*	0.324	0.0009*
MPV (fL)	0,047	0.640	0.216	0.03*	0.212	0.03*	0.210	0.04*	−0.064	0.522
PDW	0.134	0.182	0.370	0.0001*	0.228	0.02*	0.228	0.02*	−0.202	0.04*
PCT (%)	−0.007	0.942	−0.145	0.149	−0.147	0.143	−0.125	0.214	0.353	0.0003*

*CRP, C-reactive protein; WBC, White blood cell; NEU, Neutrophil; LYMPH, Lymphocyte; PLT, Platelet; MPV, Mean platelet volume; PDW, Platelet distrubition width; PCT, Plateletcrit. The correlation analyses were performed using the Spearman's correlation analysis, since data were not normally distributed. A *P*-value <0.05 was considered statistically significant.

Table 3 **s**ummarises the results of the receiver operating characteristic (ROC) curve analysis performed to evaluate the effectiveness of PIs in sepsis diagnosis compared to the control group. As visualised in Figure 1, for PLT, a cutoff point of < 231.0 103/µL provided 50% sensitivity and 74% specificity, with an area under the curve (AUC) of 0.583 (95% CI: 0.502–0.664). For MPV, a cutoff point of > 9.05 fL provided 59% sensitivity and 56% specificity, with an AUC of 0.585 (95% CI: 0.505–0.664). PDW provided 76% sensitivity and 49% specificity, with an AUC of 0.658 (95% CI: 0.582–0.733). For PCT, a threshold value of < 0.22% was determined, with 45% sensitivity and 76% specificity, resulting in an AUC of 0.590 (95% CI: 0.509–0.671).

**Table 3:** Results of ROC analysis for platelet indices in predicting sepsis compared to control.

Platelet Parameters	Cut-off point	Sensitivity %	Specificity %	Likelihood ratio	AUC	CI 95% AUC
PLT (10^3^/uL)	<231.0	50	74	1.92	0.583	0.502–0.664
MPV (fL)	>9.05	59	56	1.34	0.585	0.505–0.664
PDW	>15.8	76	49	1.49	0.658	0.582 -0.733
PCT (%)	<0.22	45	76	1.88	0.590	0.509–0.671

*PLT, Platelet; MPV, Mean platelet volume; PDW, Platelet distrubition width; PCT, Plateletcrit; AUC, Area under curve; CI, Confidence interval. Receiver operating characteristic (ROC) curve analysis was used to determine the most accurate diagnostic biomarker to differentiate between sepsis and normal. The areas under curves (AUC) were calculated and the specificity and sensitivity for the platelet count, MPV, PDW, and PCT of the most appropriate cut-off point were calculated for predicting sepsis.

**Figure 1:**
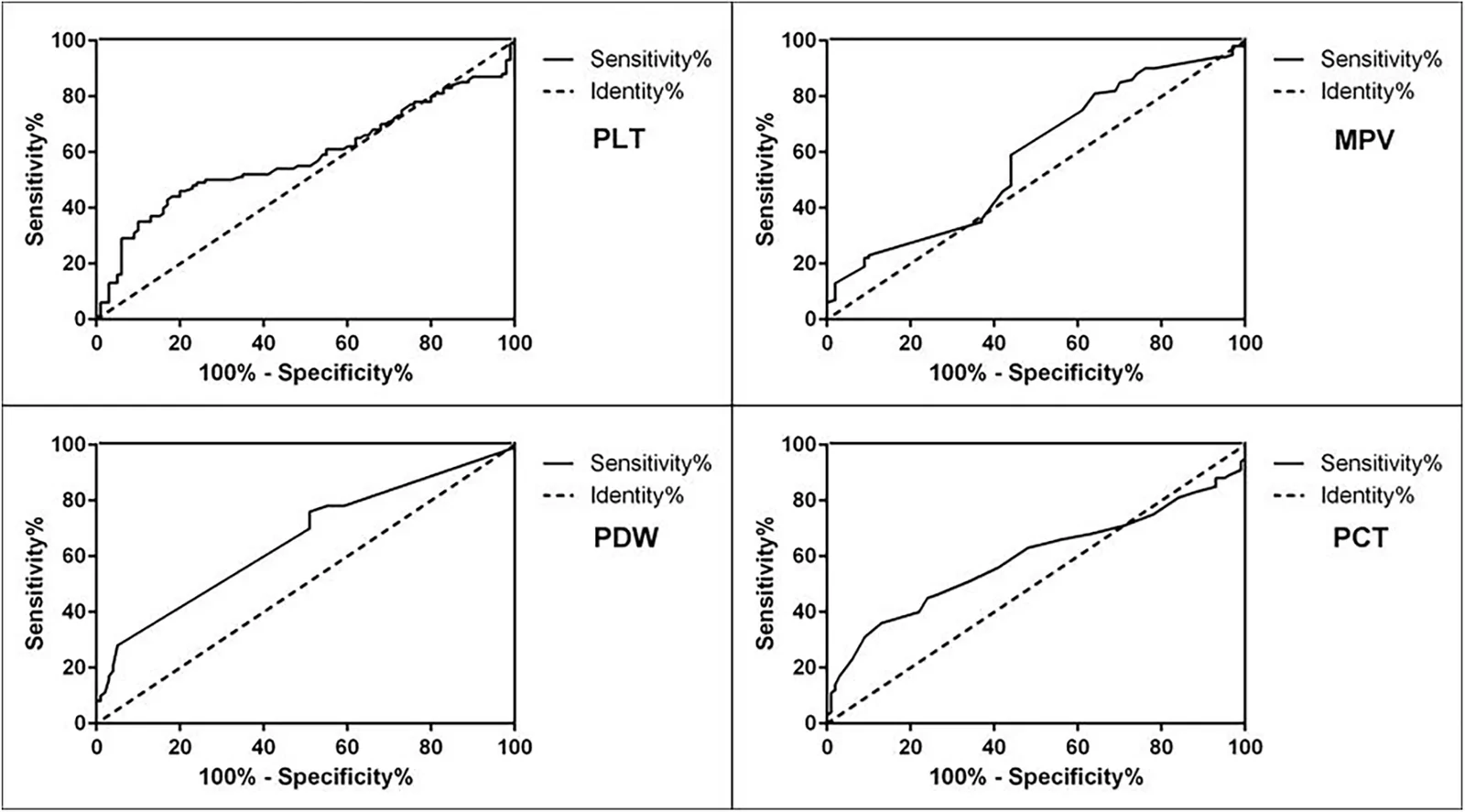
Receiver operating characteristic (ROC) curves for assessing platelet count, mean platelet volume, platelet distribution width, and plateletcrit in the detection of sepsis.

Multivariate logistic regression analysis was applied to evaluate whether significant changes in PIs were independent of the main demographic differences between groups (especially significant age difference) (Table 4). In the model, sepsis status was taken as the dependent variable; age, sex, and basic hemogram scoring parameters (WBC, HGB, PLT, MPV, and PDW) were included in the model simultaneously. As a result of the analysis, it was observed that advanced age had an independent contribution to sepsis status (OR=1.049, 95% CI: 1.025–1.074, *P* < 0.001). Among laboratory parameters, high WBC scores emerged as an independent risk factor (OR=1.278, 95% CI: 1.125–1.452, *P* < 0.001), while high HGB scores were found to have a protective effect (OR=0.732, 95% CI: 0.598–0.896, *P* = 0.002). Even after controlling for patient age, sex, Hb level, and leukocytosis, both MPV (OR=1.470, 95% CI: 1.025–2.108, *P* = 0.036) and PDW (OR=2.042, 95% CI: 1.306–3.194, *P* = 0.001) remained strong and independent predictors of sepsis. Specifically, each unit increase in the PDW score approximately doubled the probability of sepsis. This demonstrated that PDW has strong diagnostic value, regardless of the patient's age.

**Table 4:** Multivariate logistic regression analysis for independent determinants of sepsis.

Variables	Regression coefficient	Standard error	Odds Ratio (OR)	95% CI for OR	*P*-value
Age	0.048	0.012	1.049	1.025–1.074	<0.001
Gender (Male)	0.185	0.295	1.203	0.675–2.146	0.532
WBC (10^3^/µL)	0.245	0.065	1.278	1.125–1.452	<0.001
HGB (g/dL)	−0.312	0.104	0.732	0.598–0.896	0.002
PLT (10^3^/µL)	−0.005	0.004	0.995	0.987–1.003	0.215
MPV (fl)	0.385	0.184	1.470	1.025–2.108	0.036
PDW	0.714	0.228	2.042	1.306–3.194	0.001

*OR, Odds ratio; CI, Confidence interval; WBC, White blood cell; HGB, Haemoglobin; PLT, Platelet; MPV, Mean platel et volume; PDW, Platelet distribution width. Sepsis status (Control = 0, Patient = 1) was used as the dependent variable. A *P* < 0.05 value was considered statistically significant.

Figure 2 shows the ROC curves of platelet-based biomarkers evaluated in the diagnosis of sepsis. While platelet parameters alone showed limited discriminatory performance, the diagnostic accuracy of combined indices increased significantly. In particular, the WBC × PDW index showed the highest performance (AUC value: 0.815). This was followed by the PDW/PLT ratio (AUC=0.748) and the MPV/PLT ratio (AUC=0.712), respectively. According to the ROC analysis results, combined indices obtained from routine hemogram parameters have a stronger diagnostic value in predicting sepsis compared to platelet indicators used alone.

**Figure 2:**
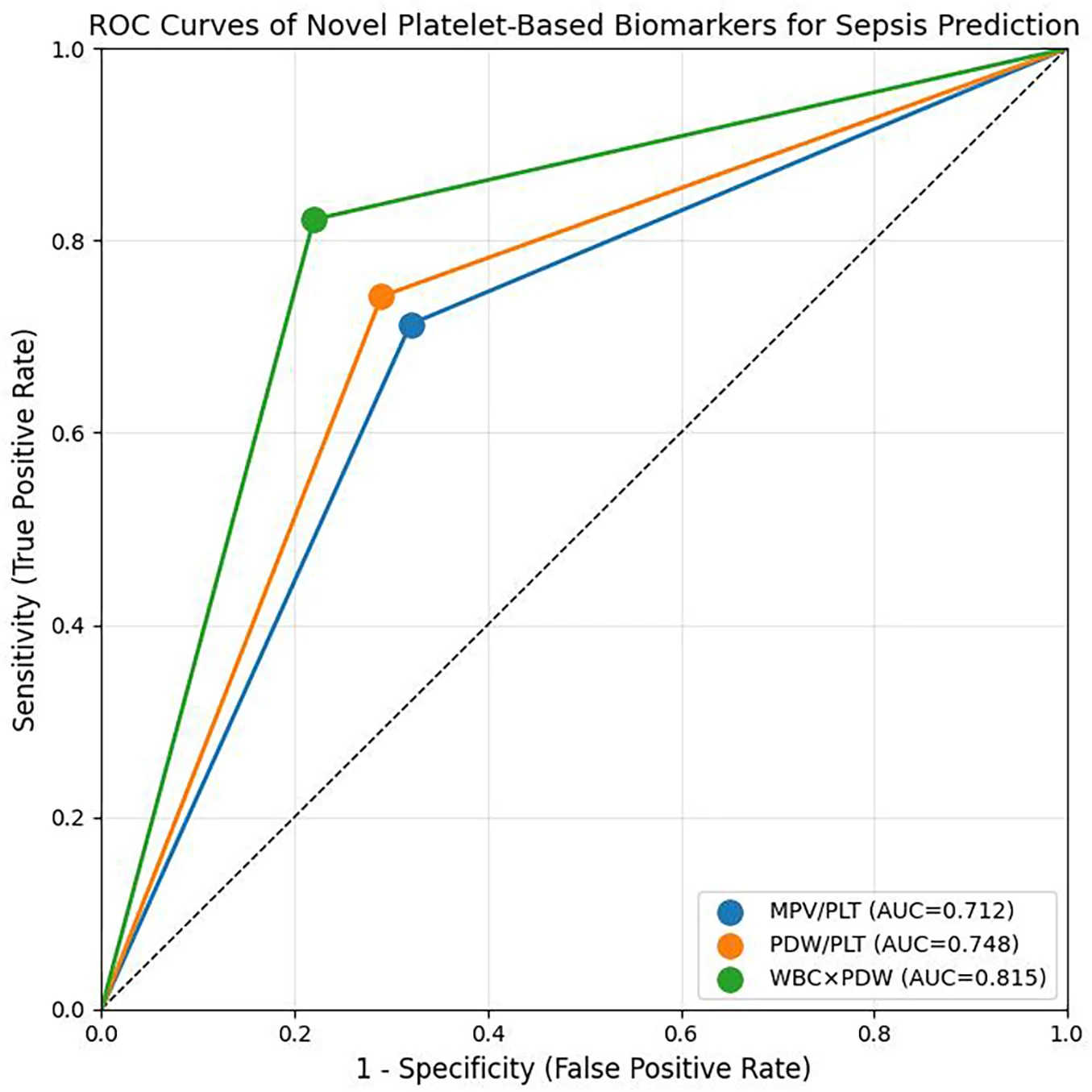
Distribution profiles of the novel combined index score between the healthy control cohort and patients with sepsis. Horizontal lines within the boxes represent the median values, the boxes represent the interquartile range (IQR), and the whiskers indicate the minimum and maximum values.

Table 5 summarises the results of ROC analyses of PIs alone and in combination with haemogram indices. ROC analysis revealed that individual platelet parameters had limited diagnostic utility for sepsis, with AUC values ranging from 0.583 to 0.658. In contrast, combined hemogram indices demonstrated significantly enhanced discriminatory power. The MPV/PLT and PDW/PLT ratios yielded AUCs of 0.712 (95% CI: 0.641–0.783) and 0.748 (95% CI: 0.680–0.816), respectively. The WBC × PDW index achieved the highest overall diagnostic performance, with an AUC of 0.815 (95% CI: 0.754–0.876, cut-off > 32.0, sensitivity 82.2%, specificity 78.0%, *P* < 0.001).

**Table 5:** ROC analysis for combined hemogram indices.

Biomarkers/Indices	Cut-off Point	Sensitivity %	Specificity %	Likelihood Ratio	AUC	95% CI for AUC	*P*-value
WBC (10^3^/uL)	>9.5	78.2	79.0	3.55	0.812	0.752–0.872	<0.001
PLT (10^3^/µL)	<231.0	50.0	74.0	1.92	0.583	0.502–0.664	0.030
MPV (fl)	>9.05	59.0	56.0	1.34	0.585	0.505–0.664	0.040
PDW	>15.8	76.0	49.0	1.49	0.658	0.582–0.733	<0.001
PCT (%)	<0.22	45.0	76.0	1.88	0.590	0.509–0.671	0.030
MPV/PLT Ratio	>0.039	71.3	68.0	2.23	0.712	0.641–0.783	<0.001
PDW/PLT Ratio	>0.068	74.2	71.0	2.56	0.748	0.680–0.816	<0.001
WBC \ PDW Index	>32.0	82.2	78.0	3.74	0.815	0.754–0.876	<0.001

**PLT, Platelet count; MPV, Mean platelet volume; PDW, Platelet distribution width; PCT, Plateletcrit; WBC, White blood cell count; AUC, Area under the curve; CI, Confidence interval; ROC, Receiver operating characteristic.

Pairwise ROC comparisons were performed using the DeLong test to verify whether the higher AUC values ​​of the combined indices reflected a genuine statistical superiority over individual markers. The results demonstrated that the diagnostic performance of the WBC × PDW index was statistically significantly superior to that of PDW alone *Δ*AUC = 0.157, 95% CI: [0.082–0.232], *p* < 0.001) and PLT alone (*p* < 0.001).

## Discussion

Although there have been advances in the medical field regarding the pathophysiology of sepsis, it remains a global health problem associated with high rates of mortality and morbidity. Early diagnosis of sepsis is life-saving. Cost and time constraints associated with traditional biomarkers (CRP and procalcitonin) make it essential to use an easier-to-apply screening tool in clinical triage. Our study has demonstrated that peripheral thrombocytopenia and bone marrow megakaryocyte responses triggered in the early stages of the pathophysiology of sepsis are clearly reflected in PIs (MPV and, in particular, PDW, which has a sensitivity of 76%) that can be measured during a complete blood count without incurring additional costs.

We found that the mean age of patients in the sepsis group was statistically significantly higher than that of the control group (66 ± 14 years versus 49 ± 21 years; *P* < 0.0001). This finding is consistent with literature data (21, 22) highlighting that advanced age is a significant risk factor for the development of sepsis, arising from age-related loss of immune function and the high burden of chronic comorbidities in elderly individuals. However, to minimise potential bias in our study, age-adjusted multivariate regression models were used in the analyses to isolate the independent effect of sepsis on haematopoiesis. Using this method, it was concluded that the changes in PIs observed in our study were attributable to the systemic inflammatory response triggered by sepsis rather than the age factor.

The current study and the research by Schupp et al. (23) both highlighted the significance of platelet dynamics in sepsis, albeit from different angles. While the current study compared PIs between sepsis patients and healthy controls, revealing lower counts and significant changes in indices, Schupp et al. (23) have found a link between a platelet count below 150  ×  10⁶/mL and an increased 30-day mortality rate and that a drop in platelet count during the course of sepsis further increases the risk of mortality by more than 25%.

The current study's findings on platelet counts in sepsis patients align with the broader literature emphasising the prognostic significance of thrombocytopenia in critically ill individuals. Various studies have shown that lower platelet counts correlate with higher rates of ICU and long-term mortality (24, 25), reinforcing the idea that platelet dynamics are crucial indicators of patient outcomes. Specifically, Claushuis et al. (26) examined 931 sepsis patients and found that both low (<50 × 10⁶/mL) and intermediate-low (50–99 × 10⁶/mL) platelet counts were associated with increased all-cause mortality. This supports the current study's findings that sepsis patients exhibit altered PIs compared to healthy controls, highlighting the critical role of platelet counts in assessing severity and prognosis. However, Claushuis et al. (26) noted stable platelet levels during the first 20 days of sepsis, without further risk stratification based on platelet decline. This contrasts with the current study's observations of significant changes in platelet parameters, suggesting that dynamic monitoring of platelet counts could provide additional insights into patient outcomes.

Gupta et al. (27) found in their similar study that MPV (11.43 ± 1.72) and PDW (21.45 ± 4.42) increased in parallel with the severity of sepsis, whilst PCT (0.5 ± 0.52) decreased. Our study examined PIs in greater detail; however, due to the small sample size, subgroup analyses for Gram-positive and Gram-negative bacteria could not be performed.

Paediatric studies also support our findings regarding the dynamic monitoring of PIs and their ability to predict mortality. Indeed, İşgüder et al. (28) demonstrated that, in paediatric patients with sepsis, both the MPV values at presentation and at 72 h, as well as the upward trend between these two time points, are strong independent risk factors for 28-day mortality. In the same study, it was found that MPV values were significantly higher and platelet counts were lower in deceased patients (*P* = 0.002) (28). The high MPV and low platelet counts observed in the sepsis group in our study are similar to the dynamic platelet changes during the sepsis process in paediatric patients reported by İşgüder et al. (28).

In our study, PDW showed the strongest positive correlation with procalcitonin (r = 0.370, *P* = 0.0001) and demonstrated the highest standalone screening potential in our ROC analysis; it provided a sensitivity of 76% at a cut-off value of >15.8 (AUC = 0.658, 95% CI: 0.582–0.733). Our findings differ somewhat from those of Lu et al. (22), who reported in their study on neonatal sepsis that traditional coagulation parameters such as prothrombin time (PT) were superior to PIs. This clinical distinction may stem from the different pathophysiological profiles of the immune responses in adults and newborns. In the adult population, our data support the views of Becchi et al. (7) and Oh et al. (17); they confirm that changes in MPV and PDW occur in the early stages of sepsis and may serve as valuable diagnostic indicators.

We found higher MPV in sepsis patients (*P* = 0.04) and lower platelet counts (*P* = 0.03), identifying key diagnostic cut-offs. In contrast, Oh et al. (17) focused on the MPV to platelet count ratio as a prognostic marker for early mortality in severe sepsis patients receiving early goal-directed therapy, finding that higher ratios on admission and at 24 h significantly correlated with increased 28-day mortality risk. While our study highlights individual PIs, Oh et al. (17) emphasise the MPV/PLT ratio as a valuable prognostic tool, suggesting different clinical applications in sepsis management.

Recent studies in the literature have investigated the superiority of combined blood count-based indices over individual parameters in the diagnosis and monitoring of sepsis. Meta-analyses by Zhu et al. (29) have demonstrated that combined biomarkers such as the NLR, systemic inflammatory index and MPV/PLT possess high diagnostic accuracy in sepsis; this finding is consistent with our study, where whilst individual parameters were borderline, the combined index WBC × PDW demonstrated the highest diagnostic power (AUC = 0.815). Zhao et al. (30) identified the high success of platelet-based combined indices (D-dimer/PLT) in predicting mortality (AUC=93.9); the high PDW and low PLT levels we identified as independent predictors of sepsis in our study support their potential for use as cost-effective and rapid early screening tools in clinical practice. In their meta-analysis, Tong and colleagues (31) noted that the MPV/PLT ratio demonstrated a low sensitivity of 55% in adults with sepsis and that, for this reason, it could only serve as a ’screening criterion’ in settings where resources are limited. In contrast, our study evaluated stronger combinations of routine blood count parameters and found that the WBC × PDW index, when used in combination, was identified as a diagnostic test with higher discriminatory power (AUC = 0.815).

This study examines the utility of routine haematological parameters in the early diagnosis of patients with sepsis. In the sepsis group, a marked increase in WBC count and an increase in neutrophil count were observed, while the lymphocyte count was lower. Platelet counts were decreased, and a higher MPV was observed in the sepsis group. One of the most important findings of this study is that the combination of complete blood count parameters demonstrated diagnostic superiority compared to the use of platelet parameters alone. Among all the biomarkers evaluated, the WBC × PDW index demonstrated the strongest discriminatory ability for sepsis (AUC = 0.815); this index outperformed both the PDW/PLT ratio (AUC = 0.748) and the MPV/PLT ratio (AUC = 0.712). These findings suggest composite inflammatory PIs may be useful for early sepsis detection as simple, cost-effective, and readily available tools. While the WBC × PDW index showed promising results in a retrospective analysis, it requires further prospective validation before clinical use. However, the WBC × PDW index showed promising results in this retrospective study, a further prospective study is necessary before its clinical application.

### Limitations of the study

This study acknowledges several limitations that may influence the interpretation of its findings. Since the results obtained are based on a limited number of patients, they may not be generalisable to larger patient populations. Stringent exclusion criteria could introduce selection bias, potentially omitting critical patient subgroups, such as those in intensive care or experiencing septic shock. Additionally, the study did not thoroughly assess the effects of comorbidities or concurrent medications on platelet parameters, which could confound the results. The reliance on laboratory tests at specific time points may limit the understanding of dynamic changes in PIs during sepsis progression. Furthermore, the specificity of some platelet parameters is relatively low, indicating the need for further validation through larger, multicenter studies to improve diagnostic accuracy and clinical applicability. A key limitation is the small sample size and single-centre, retrospective design, impacting the statistical power of the diagnostic models. This small sample size hindered robust subgroup analyses (e.g., Gram-positive/negative or sepsis/septic shock) and is the primary reason for the lower AUC values (0.58–0.65) observed in the ROC analysis compared to larger studies in existing literature. The absence of an age-matched control group resulted in a significant age difference, with sepsis patients being older than healthy controls (66 ± 14 vs. 49 ± 21 years; *P* < 0.0001). Although multivariate logistic regression analyses were performed to account for this difference, age-related confounding factors may still influence hematological parameters. Future prospective designs utilising dynamically balanced age cohorts are recommended to completely eliminate any potential age-associated compounding vectors on baseline platelet morphology.

## Data Availability

The original contributions presented in the study are included in the article/Supplementary Material, further inquiries can be directed to the corresponding author.
